# Diet and Lifestyle Quality in Australian Females with Endometrial Cancer at Diagnosis: Insights from the Fertility-sparing Management for Early Endometrial Cancer (FeMMe) Trial

**DOI:** 10.1016/j.cdnut.2025.107572

**Published:** 2025-10-11

**Authors:** Ruqaiya Al Ramadhani, Monika Janda, Mary Playdon, Andreas Obermair

**Affiliations:** 1 Centre for Health Services Research, Faculty of Health, Medicine and Behavioural Sciences, The University of Queensland, Woolloongabba, QLD, Australia; 2 Department of Nutrition and Integrative Physiology, College of Health, The University of Utah, Salt Lake City, UT, United States; 3 Cancer Control and Population Sciences, Huntsman Cancer Institute, The University of Utah, Salt Lake City, UT, United States; 4 Queensland Centre for Gynaecological Cancer Research, Faculty of Health, Medicine and Behavioural Sciences, The University of Queensland, Herston, QLD, Australia

**Keywords:** endometrial cancer, diet and lifestyle, body composition, glycemic control, quality of life, mental health

## Abstract

**Background:**

Diet, lifestyle, and body composition may impact quality of life (QoL) and health outcomes in females with endometrial cancer (EC). Many seek guidance on improving prognosis, treatment outcomes, and QoL through nutrition and other modifiable lifestyle factors.

**Objectives:**

This study aims to investigate diet and lifestyle quality in FeMMe trial participants and associations with glycemic control, body composition, QoL, and mental health before treatment.

**Methods:**

Baseline data from the multicenter, 3-arm, phase II FeMMe trial (NCT01686126), which enrolled females with obesity and early-stage EC or complex endometrial hyperplasia with atypia undergoing conservative hormonal therapy. The Mediterranean diet score (MDS), World Cancer Research Fund/American Institute for Cancer Research (WCRF/AICR), and Extended Healthy Lifestyle Index (EHLI) scores were derived from self-reported dietary and lifestyle data. QoL, depression, and anxiety were measured using validated tools. Anthropometry and body composition (height, weight, hip, and waist circumference) were measured by a study nurse. Glycemic control was assessed using blood samples, utilizing the Homeostatic Model Assessment for Insulin Resistance as the measurement metric. Bonferroni correction applied for multiple comparisons; nominal associations defined as *P* < 0.05 but ≥ 0.001.

**Results:**

Approximately 40.7%, 31.3%, and 34.7% of females had good diet and/or lifestyle quality according to the MDS, WCRF/AICR, and EHLI, respectively. We found that higher WCRF/AICR scores, expressed per standard deviation (SD) increase, were nominally associated with a better overall QoL score (*β*_*perSD*_ = 4.45, 95% confidence interval (CI): 0.30, 8.61, *P* = 0.036), and with higher anxiety and depression scores (*β*_*perSD*_ = 0.60, 95% CI: 0.09, 1.11, *P* = 0.02). For the EHLI, each SD increase was nominally associated with 8% reduction in insulin resistance (Exp(*β*_*perSD*_) = 0.92, 95% CI: 0.85, 0.996, *P* = 0.039).

**Conclusions:**

Among females with EC and BMI ≥ 30 kg/m^2^, healthier lifestyle patterns were associated with better QoL and insulin sensitivity, but also higher anxiety and depression.

## Introduction

Endometrial cancer (EC) is a major health concern among females with obesity [1]. For every 5 unit increase in BMI, risk of developing EC doubles [2]. Obesity is also associated with poorer EC prognosis including greater risk of cancer recurrence and lower survival rates [3]. Additionally, females with a BMI of 30 kg/m^2^ or higher are at elevated risk of other obesity-related health conditions such as diabetes mellitus, hypertension, and cardiovascular diseases [4]. These comorbidities complicate EC treatment and contribute to poorer outcomes [5]. Therefore, it is imperative to identify interventions with potential to improve patient outcomes after an EC diagnosis.

There are currently no specific guidelines on diet and lifestyle changes to improve outcomes for EC survivors [2]. A few studies suggest that lifestyle interventions that promote physical activity and better quality diet can contribute to successful weight loss and improved glycemic control, physical functioning, and quality of life (QoL) among EC survivors, although these studies had poor participant retention, and did not target females with severe obesity that make up 19%–36% of patients with EC [[6], [7], [8], [9]]. Patients with EC with obesity encounter significant challenges due to their body weight, including higher rates of comorbidities and risk of surgical complications [10]. Surgical hysterectomy is the most common treatment of EC, but severe obesity may preclude surgical eligibility [11]. Obesity pharmacotherapy such as glucagon-like peptide-1 receptor agonists lead to significant weight loss, but discontinuation rates are high and it remains unclear how these medications affect muscle mass and cancer outcome [12]. Lifestyle modification to reduce BMI can assist patients to become eligible for surgical procedures. Furthermore, patients with cancer, including those with EC, often experience psychological distress such as depression and anxiety, which can further impact their QoL [13]. Research has shown that adopting healthy dietary patterns like the Mediterranean diet and engaging in regular physical activity are associated with reduced depressive symptoms and anxiety levels in various populations [14,15]. However, there is a lack of studies focusing on females with obesity and EC, who may have more difficulty than other patients with cancer in adopting such behaviors due to multiple previous weight loss attempts and traumatic experiences in the past [16,17]. Overall, although there is some evidence suggesting that healthy lifestyle practices and weight loss may influence the well-being of EC survivors; more research is needed to establish clear associations and guide supportive care for EC survivors.

This study aimed to address this research gap by investigating the diet and lifestyle quality of females who participated in the FeMMe trial. This trial is the only known Australian study and one of very few globally to collect validated dietary data from patients with EC undergoing conservative hormonal treatment. The trial focused on females with a BMI ≥ 30 kg/m^2^ who were ineligible for surgery due to elevated surgical risk, representing a clinically important yet under-researched subgroup [18]. Leveraging FeMMe’s comprehensive pretreatment assessments, we examined associations between lifestyle factors and glycemic control, body composition, QoL, and mental health in FeMMe trial participants before treatment. We hypothesized that healthier lifestyle behaviors would be associated with more favorable glycemic control, improved body composition, and better QoL and mental health outcomes. The cross-sectional nature of the data precludes establishing temporality. Therefore, our estimates should be interpreted as associations.

## Methods

### Study design and population

We leveraged baseline data from the multicenter, 3-arm, phase II, randomized controlled clinical FeMMe trial (NCT01686126). The study was approved by Human Research Ethics Committees at all participating institutions in Australia and New Zealand; informed consent was obtained from all participants before randomization. Full details of the study protocol and primary outcomes were described previously [18,19].

Briefly, FeMMe included 165 females who were aged ≥ 18 y, with histologically confirmed complex endometrial hyperplasia with atypia or stage 1 endometrioid adenocarcinoma, BMI ≥ 30, myometrial invasion of < 50% on MRI for patients with EC, and cancer antigen 125 ≤ 30U/mL, who were at risk of surgical complications or ineligible for surgery due to obesity, comorbidities, or retention of fertility. Females were eligible if they had no contraindications to levonorgestrel-releasing intrauterine device (LNG-IUD) or metformin, a negative pregnancy test, had not used metformin for more than 2 years prior to enrollment, and had either never used an LNG-IUD or had one inserted less than 6 weeks before enrollment.

Baseline data were collected using questionnaires and included information on sociodemographic characteristics, medical history including comorbidities used to calculate the Charlson Comorbidity Index [20], QoL, and health behavioral data, as well as objectively measured height, weight, and waist and hip circumferences. Blood and endometrial tissue for biomarker studies were also collected at baseline. The baseline questionnaire also collected information on lifestyle over the prior 12 mo before the diagnosis of EC. Dietary intake data were collected using the Dietary Questionnaire for Epidemiological Studies Version 2 (DQES v2), developed by Cancer Council Victoria, Australia [21]. This semiquantitative food frequency questionnaire (FFQ) was originally designed for the Melbourne Collaborative Cohort Study, which included adults aged 40–69 y [21]. The DQES v2 has been validated in Australian populations through comparisons with weighed food records and biomarkers, demonstrating acceptable validity for key nutrients [22,23]. Reproducibility has also been assessed, with nutrient estimates correlating well with those from other validated FFQs [22]. Although it has not been specifically validated in females with obesity or early-stage cancer, it has been used in studies involving adults with varying BMI and health conditions, supporting its suitability for our study population [[24], [25], [26], [27]]. Physical activity was measured using the Active Australia Survey, which has demonstrated acceptable reliability and validity for use in population health research in Australian adults [28,29]. FeMMe participants were included in the current analysis if they completed the FFQ (*n =* 157). After excluding females with implausible energy intake (< 2568 kJ or > 17,031 kJ) (∼ < 614 kcal or > 4070 kcal) (*n =* 6) based on ±3 SDs of log-transformed mean energy intake, those missing baseline demographic data (*n =* 1) and those with unknown level of physical activity (*n =* 3), 150 females provided data for the diet-only analysis and 147 females for diet and lifestyle analysis. Supplemental Figure 1 illustrates the flowchart of study participants.

### Diet and lifestyle quality scores

The FFQ and Active Australia Study were used to calculate the Mediterranean diet score (MDS), the World Cancer Research Fund/American Institute for Cancer Research guidelines score (WCRF/AICR), and the Extended Healthy Lifestyle Index (EHLI). These indices were selected based on their established associations with reduced risk of EC [[30], [31], [32]]. The MDS is based on intake of specific foods/food groups only. WCRF/AICR assesses adherence to cancer prevention and survival lifestyle recommendations that include both diet and physical activity. Extended Healthy Lifestyle Index (EHLI) comprises diet and lifestyle behaviors but is not specifically focused on cancer. The derivation methods and validation of all three indices have been described previously [[33], [34], [35]]. Briefly, the MDS has demonstrated predictive validity through its consistent associations with health outcomes across populations [36], and the WCRF/AICR score has shown predictive validity for chronic disease and cancer outcomes in large cohort studies using a standardized scoring system [37]. Although the original HLI is more extensively validated, EHLI was chosen for its additional dietary components that better reflect current cancer prevention guidelines. Although formal validation is limited, it has demonstrated construct validity in cancer epidemiology [35].

The following sections provide a detailed overview of how each index was constructed and applied in this study.

#### Mediterranean diet score

MDS comprises 9 dietary components: fruit, vegetables, cereals, legumes, fish, dairy products, meat, monounsaturated to saturated fatty acids (SFA) ratio, and alcoholic beverages [33]. Each component is scored 0 or 1 based on the median intake (measured in grams/day (g/d)). Beneficial components (fruit, vegetables, cereals, legumes, fish, and a high monounsaturated to saturated fat ratio) receive a score of 1 for at or above the median intake, and 0 for below. Conversely, for dairy and meat, a score of 1 is assigned for below-median intake, and 0 for above. For alcohol, 1 point is awarded to females consuming 5 g to <25 g of ethanol per day and 0 for > 25 g or 0 g to < 5 g of alcohol intake. The total MDS can range between 0 and 9 with higher scores indicating better adherence to a Mediterranean diet. For details of MDS score derivation, see Supplemental Table 1.

#### World Cancer Research Fund/American Institute for Cancer Research

The WCRF/AICR score is a dietary and lifestyle index [34] based on the following 9 recommendations: *1*) optimizing BMI (kg/m^2^), *2*) physical activity (minutes/day of moderate or vigorous intensity activity), *3*) avoiding energy dense food (measured in kcal/g) and sugary drinks (g/d), *4*) eating more plant-based food (g/d), *5*) limiting red and processed meat intake (g/wk), *6*) limiting alcohol (g/d), and *7*) reducing salt intake (g/d), *8*) meeting nutritional needs through diet alone rather than supplements, and *9*) special recommendations, including breastfeeding for ≥ 6 mo. Each recommendation is scored as 1 for low-risk behavior, 0.5 for moderate risk, and 0 for high risk. We modified the WCRF score by excluding the salt intake, supplements, and breastfeeding duration components, because no data were collected for those factors; therefore, the total score in our study could range from 0 to 6. For details of WCRF/AICR score derivation, see Supplemental Table 2.

#### Extended Healthy Lifestyle Index

EHLI is an extension to the original HLI with 4 additional dietary recommendations [35]. The original HLI comprises 5 components: *1*) smoking status, *2*) alcohol intake (g/d), *3*) physical activity (minutes/week of moderate or vigorous intensity activity), *4*) BMI (kg/m^2^), and *5*) fruit and vegetable consumption (g/d) [38]. The added components to EHLI are: *1*) drinks that promote weight gain (g/d), *2*) red and processed meat (g/d), *3*) unprocessed grains and/or legumes (g/d), and *4*) dairy products (g/d). Each recommendation was scored from 0 (noncompliance) to 1 (full compliance) and 0.5 for partial compliance. The total score can range from 0 to 9. The details of EHLI score derivation are presented in Supplemental Table 3.

### Outcomes assessment

#### Body composition

Weight (kg), height (cm), waist, and hip circumference (cm) were measured by a study nurse. Body mass index (BMI, kg/m²) and waist-to-hip ratio were calculated.

#### Glycemic control parameters

Glycated hemoglobin (HbA1c, %), fasting glucose (mmol/L), and fasting insulin (nmol/L) were measured from blood samples collected by the study nurse or pathology providers. To quantify insulin resistance, the clinically validated HOMA-IR method was used [39,40], calculated as: HOMA-IR = (fasting glucose × fasting insulin) / 22.5, where higher scores reflect greater insulin resistance).

#### Quality of life

The Functional Assessment of Chronic Illness Therapy (FACIT) questionnaire was used for QoL assessment [41]. It comprises 5 QoL domains including physical well-being (7 items), social well-being (7 items), emotional well-being (6 items), functional well-being (7 items), and additional concerns specific to EC (16 items). Each item is scored 0–4, where 0 = “Not at all,” 1 = “A little bit,” 2 = “Somewhat,” 3 = “Quite a bit,” 4 = “Very much,” with a total score of 172. Negative questions were reverse scored. Higher scores of FACIT reflect better QoL. In the case of missing data, subscale scores were prorated if > 50% of items were answered. Similarly, the overall FACIT score was calculated if > 80% of questions were answered, as per the FACIT manual [42].

#### Depression and anxiety scores

The Hospital Anxiety and Depression Scale (HADS) was used to measure anxiety and depression [43]. The HADS overall score is a 14-item self-report measure, with 7 items forming a depression and 7 an anxiety subscale. Each item is rated on a 4-point scale, ranging from 0 to 3 (3 indicates higher symptom frequency). Total scores for each subscale range from 0 to 21, categorized as: normal (0–7), mild (8–10), moderate (11–14), and severe (15–21). Missing answers were handled using the same method as the QoL score (acceptable response rate > 50% for subscale scores and > 80% for overall HADS score).

### Statistical analysis

Descriptive data (*N* and proportion for categorical variables and mean ± SD for continuous variables) were used to summarize participant characteristics, including sociodemographic, family, and clinical history, overall and by tertiles of MDS, WCRF/AICR, and EHLI scores. Chi-square tests for categorical variables, and analysis of variance or Kruskal–Wallis tests for continuous variables compared participant characteristics according to diet and lifestyle score tertiles. Adherence to dietary and lifestyle recommendations was assessed using the total scores from each index, with tertile 3 representing good overall adherence. Additionally, for individual dietary and lifestyle components, adherence was defined based on whether participants met the maximum score criteria for that item, as outlined in Supplemental Tables 1–3. These item-level adherence proportions are reported in Table 1. To assess for correlations and agreements between the MDS, WCRF/AICR, and EHLI, Spearman correlation tests (continuous scores) and weighted kappas (tertiles) were used, respectively.

**TABLE 1:** Baseline characteristics of FeMMe participants overall (*N =* 150).

Baseline characteristics	*N* (%)
Age (y)	
<50	51 (34.0)
≥50	99 (66.0)
Race	
White	103 (68.7)
Non-white/others	47 (31.3)
Education level	
High school or less	80 (53.7)
TAFE or diploma or university	69 (46.3)
Missing	1
Income (annual)	
Low (<$10,000–$20,000)	35 (23.3)
Middle (>$20,000–$60,000)	57 (38.0)
High (>$60,000)	51 (34.0)
Missing	7
Marital status	
Partner	88 (59.1)
No partner	61 (40.9)
Missing	1
Menopausal status	
Pre	61 (40.7)
Post	88 (59.3)
Smoking	
Never	82 (54.7)
Former	54 (36.0)
Current	14 (9.3)
Family history of cancer	
No	33 (24.6)
Yes	101 (75.4)
Missing	16
Diabetes	
No	104 (69.3)
Yes	46 (30.7)
Depression	
No	128 (85.3)
Yes	22 (14.7)
Anxiety	
No	138 (92.0)
Yes	12 (8.0)
Comorbidities^1^	
0	52 (34.7)
1+	98 (65.3)
Physical activity	
No	114 (77.6)
Yes	33 (22.5)
Missing	3
Dietary items with adherence	
Fruit and nuts intake (MDS)	75 (50.0)
Fruit and nuts intake (WCRF/AICR)	—
Fruit and nuts intake (EHLI)	—
Vegetables intake (MDS)	74 (49.3)
Vegetables intake (WCRF/AICR)	—
Vegetables intake (EHLI)	—
Fruit and vegetables intake (MDS)	—
Fruit and vegetables intake (WCRF/AICR)	64 (42.7)
Fruit and vegetables intake (EHLI)	64 (42.7)
Total fiber intake (MDS)	—
Total fiber intake (WCRF/AICR)	31 (20.7)
Total fiber intake (EHLI)	—
Refined grains intake (MDS)	—
Refined grains intake (WCRF/AICR)	50 (33.3)
Refined grains intake (EHLI)	—
Whole/unprocessed grains intake (MDS)	—
Whole/unprocessed grains intake (WCRF/AICR)	—
Whole/unprocessed grains intake (EHLI)	19 (12.7)
Legumes intake (MDS)	75 (50.0)
Legumes intake (WCRF/AICR)	Included in the fruit and vegetable category
Legumes intake (EHLI)	Included in the whole grains category
Cereals intake (MDS)	75 (50.0)
Cereals intake (WCRF/AICR)	—
Cereals intake (EHLI)	—
Fish and seafood intake (MDS)	75 (50.0)
Fish and seafood intake (WCRF/AICR)	—
Fish and seafood intake (EHLI)	—
Dairy products intake (MDS)	75 (50.0)
Dairy products intake (WCRF/AICR)	—
Dairy products intake (EHLI)	129 (86.0)
Red and processed meat intake (MDS)	75 (50.0)
Red and processed meat intake (WCRF/AICR)	5 (3.3)
Red and processed meat intake (EHLI)	7 (4.7)
Sugary drinks intake (MDS)	—
Sugary drinks intake (WCRF/AICR)	28 (18.0)
Sugary drinks intake (EHLI)	67 (44.7)
Energy dense food intake (MDS)	—
Energy dense food intake (WCRF/AICR)	73 (57.0)
Energy dense food intake (EHLI)	—
Monounsaturated to saturated fat ratio (MDS)	71 (47.3)
Monounsaturated to saturated fat ratio (WCRF/AICR)	—
Monounsaturated to saturated fat ratio (EHLI)	—
Alcohol intake (MDS)	30 (20.0)
Alcohol intake (WCRF/AICR)	109 (72.7)
Alcohol intake (EHLI)	108 (72.0)
Good diet quality (MDS)^2^	61 (40.7)
Good diet/lifestyle quality (WCRF/AICR)^2^	46 (31.3)
Good diet/lifestyle quality (EHLI)^2^	51 (34.7)
Glycemic control parameters	
HbA1c (%), median (IQR)	5.8 (1.0)
Fasting insulin (nmol/L), median (IQR)	19.0 (20.0)
Fasting glucose (mmol/L), median (IQR)	5.9 (1.9)
HOMA-IR, median (IQR)	5.5 (5.6)
Body composition	
BMI (kg/m^2^), mean (SD)	47.5 (9.3)
Waist (cm), mean (SD)	129.7 (17.0)
Hip (cm), mean (SD)	144.9 (18.9)
Waist/hip ratio, mean (SD)	0.9 (0.1)
Quality of life (FACIT score)	
Physical, mean (SD)	20.7 (28.0)
Social, mean (SD)	21.0 (5.9)
Emotional, mean (SD)	16.8 (4.7)
Functional, mean (SD)	16.3 (5.5)
Additional, mean (SD)	46.4 (10.9)
General, mean (SD)	122.4 (23.6)
Depression and anxiety scores (HADS)	
Anxiety score, mean (SD)	12.8 (3.0)
Depression score, mean (SD)	14.7 (2.2)
Overall, mean (SD)	27.5 (4.6)

Abbreviations: BMI, body mass index; EHLI, Extended Healthy Lifestyle Index; FACIT, Functional Assessment of Chronic Illness Therapy; FeMMe, Fertility-sparing Management for Early Endometrial Cancer; HADS, Hospital Anxiety and Depression Scale; HbA1c, glycated hemoglobin; MDS, Mediterranean diet score; TAFE, Technical and Further Education; WCRF/AICR, World Cancer Research Fund/American Institute for Cancer Research.

1 Based on the Charlson Comorbidity Index score.

2 Good diet/lifestyle quality was defined as scoring in the top tertile of the respective index.

Multivariable linear regression models were used to examine the association between diet and lifestyle quality scores and health outcomes. To standardize the dietary and lifestyle quality scores, a z-transformation was performed. This approach was chosen to facilitate comparability across different diet quality indices and outcome measures, which are on varying scales. The β coefficients represent the change in the outcome variable for each SD increase in the scores. The selection of models was guided by directed acyclic graphs (DAGs) (Supplemental Figure 2) [44]. The models were built independently for each score. MDS models were adjusted for age (continuous), total energy intake (continuous), education level (high school or less / Technical and Further Education (TAFE) or diploma or university), marital status (partner / no partner), smoking (never / former / current), comorbidities (0 / 1+), physical activity (continuous, minutes/week), and BMI. The WCRF/AICR and EHLI models did not adjust for BMI, physical activity, or smoking status (EHLI) because these factors were already included in the score calculations. Additionally, for models where BMI is the outcome variable, regression analysis was not performed for the exposures WCRF/AICR and EHLI, as BMI was already incorporated into the score formulation of these indices. To account for measurement error and ensure consistency across dietary indices, we adjusted for total energy intake (kcal/d) in all models. This is particularly important as MDS and EHLI do not explicitly include energy intake, whereas WCRF/AICR only indirectly considers it [45]. Other variables that were considered in the models but ultimately removed included income, associated with education level and marital status, and diabetes, as it was associated with comorbidities. We employed multivariable regression models to reduce the risk of confounding; however, given the lack of temporality in cross-sectional data, these estimates cannot isolate the independent effect of diet and lifestyle quality on patient outcomes. We assessed the assumptions of linear regression, including linearity and homoscedasticity, through visual inspection of residual plots. The normality of residuals was evaluated using both graphical methods (Q–Q plots and histograms) and the Shapiro–Wilk test. For outcomes with non-normally distributed residuals (HbA1c, fasting insulin, fasting glucose, HOMA-IR), we applied log transformation before modeling, and estimates were exponentiated back to the original scale. In these models, the exponentiated log β coefficients reflect the percent change in the dependent variable for each percent change in the independent variable. An exploratory subgroup analysis for the association between the WCRF/AICR score and the overall anxiety and depression (HADS) scores was performed using multivariable linear regression models. To account for multiple comparisons, we applied the Bonferroni correction. Given that we examined three dietary and/or lifestyle quality indices and 17 outcomes, resulting in 51 comparisons, the threshold for stronger evidence was adjusted to 0.05/51 ≈ 0.001. We report all *P* values and corresponding confidence intervals (CIs). *P* values < 0.001 were considered to provide stronger evidence against the null hypothesis. Findings with *P* values between 0.001 and 0.05 were interpreted as nominal associations, contributing to a more nuanced interpretation. To further support this approach, we avoided binary classifications of results based solely on statistical significance thresholds. All analysis was performed using SAS version 9.4 (SAS Institute Inc.).

## Results

### Baseline characteristics (*n* = 150)

The majority of FeMMe trial participants were 50 y or older (*n =* 99, 66%), of white race (*n =* 103, 69%), with a high school or less education (*n =* 80, 54%), middle to high annual income (*n =* 108, 72%), postmenopausal (*n =* 88, 59%), living with a partner (*n =* 88, 59%), never smoked (*n =* 82, 55%), physically inactive (*n =* 114, 78%), had ≥ 1 comorbidity (*n =* 98, 65%), and a family history of cancer (*n =* 101, 75%) (Table 1). In accordance with the inclusion criterion of BMI 30+, they had high mean body composition measurements, including BMI (47.5 ± 9.3), waist circumference in cm (129.7 ± 17.0), and waist-to-hip ratio (0.9 ± 0.1). They had high median glycemic parameters (HbA1c, fasting glucose, fasting insulin, and HOMA-IR). Mean QoL scores in the emotional, functional, and EC-specific (labeled as “Additional”) domains were generally low, whereas mean mental health scores showed mostly moderate anxiety and depression. In terms of dietary and lifestyle adherence, 40.7% of participants were classified as having good adherence according to the MDS, 31.3% according to the WCRF/AICR, and 34.7% according to the EHLI, based on scoring in the highest tertile of each index. Adherence to individual dietary components, based on maximum score criteria, is also presented. All data are summarized in Table 1.

### Characteristics of females in the FeMMe trial by diet/lifestyle quality

In bivariate analyses, females with better baseline diet and lifestyle quality (tertile 3) compared with those with poor diet and lifestyle quality (tertile 1), were more likely to be older, postmenopausal, with 1 or more comorbidities, and were less likely to be of white race. Females with better diet/lifestyle scores had lower fasting insulin and insulin resistance (HOMA-IR). There were some inconsistencies in the direction of bivariate associations for the three different diet/lifestyle score tertiles, with few reaching statistical significance (Supplemental Tables 4–6). For example, females in tertile 3 (compared with tertile 1) were more likely to have a higher educational level (TAFE or diploma or university) and no family history of cancer (MDS). Conversely, the opposite trend was observed for the WCRF/AICR and EHLI scores. Regarding smoking, females in tertile 3 (compared with tertile 1) were more likely to have never smoked (EHLI), less likely to have never smoked but more likely to have a former smoking history (MDS), and showed no difference for WCRF, though there were more former smokers and fewer current smokers. Additionally, for MDS, females in the highest tertile were more likely to have a partner, report lower emotional and functional QoL, and were less likely to experience anxiety or anxiety and depression overall. In contrast, females in tertile 3 of WCRF had higher general QoL and higher levels of anxiety, as well as overall anxiety and depression, compared with those in tertile 1, with no differences observed for EHLI.

Table 2 presents the results from the multivariable regression models, reflecting the change in the β coefficient associated with each 1-SD increase in the three diet/lifestyle scores. Associations differed across the diet and lifestyle scores. A 1-SD increase in the WCRF/AICR dietary score was nominally associated with a 4.45-point increase in global QoL (95% CI: 0.30, 8.61; *P =* 0.036) and a 0.60-point increase in anxiety and depression total scores (95% CI: 0.09, 1.11; *P* = 0.02). A 1-SD increase in the EHLI score was nominally associated with an 8% decrease in insulin resistance (HOMA-IR) (95% CI: 0.85, 0.996; *P =* 0.039).

**TABLE 2:** Associations between MDS, WCRF, and EHLI scores and glycemic control, body composition, QoL, and depression and anxiety scores in FeMMe participants at baseline (*N =* 150).

Baseline characteristics	*N*	MDS score *N =* 150, mean (SD) = 4.2 (1.6)			*N*	WCRF score *N =* 147, mean (SD) = 2.0 (0.7)			*N*	EHLI score *N =* 147, mean (SD) = 3.8 (1.2)		
		*β*^1^	95% CI	*P* value		*β*^1^^,^^2^	95% CI	*P* value		*β*^1^^,^^3^	95% CI	*P* value
Glycemic control												
HbA1c (%)	149	1.00	0.99, 1.02	0.71	146	1.00	0.98, 1.01	0.52	146	0.99	0.98, 1.00	0.06
Fasting insulin (nmol/L)	135	0.96	0.89, 1.03	0.29	132	0.96	0.89, 1.04	0.33	132	0.93	0.86, 1.01	0.07
Fasting glucose (mmol/L)	141	1.00	0.98, 1.02	0.89	138	1.00	0.98, 1.02	0.80	138	0.99	0.97, 1.01	0.18
HOMA-IR	133	0.97	0.90, 1.05	0.45	130	0.97	0.90, 1.05	0.48	130	0.92	0.85, 0.996	0.039
Body composition												
BMI^4^ (kg/m^2^)	150	0.29	–1.29, 1.87	0.72	147	—	—	—	147	—	—	—
Waist (cm)	142	0.99	–0.69, 2.66	0.25	139	–0.92	–4.05, 2.21	0.56	139	–1.54	–4.52, 1.44	0.31
Hip (cm)	142	0.28	–0.98, 1.54	0.66	139	–0.88	–4.20, 2.45	0.60	139	–0.79	–3.93, 2.35	0.62
Waist/hip ratio	142	0.005	–0.01, 0.02	0.47	139	–0.002	–0.02, 0.01	0.80	139	–0.01	–0.02, 0.01	0.33
Quality of life (FACIT score)												
Physical	148	0.01	–0.94, 0.96	0.98	146	0.53	–0.44, 1.50	0.28	146	0.42	–0.51, 1.34	0.37
Social	147	–0.07	–1.06, 0.93	0.90	145	1.01	–0.004, 2.03	0.05	145	0.07	–0.91, 1.01	0.89
Emotional	147	–0.37	–1.13, 0.39	0.34	145	0.55	–0.24, 1.33	0.17	145	0.50	–0.24, 1.04	0.18
Functional	148	–0.44	–1.48, 0.60	0.40	146	0.98	–0.09, 2.04	0.07	146	0.72	–0.30, 1.51	0.17
Additional	148	–0.21	–1.52, 1.94	0.81	146	0.97	–0.79, 2.72	0.28	146	0.64	–1.03, 2.31	0.45
General	148	–0.42	–4.56, 3.72	0.84	146	4.45	0.30, 8.61	0.036	146	3.17	–1.09, 7.44	0.14
Depression and anxiety scores (HADS)												
Anxiety score	148	–0.02	–0.41, 0.38	0.93	146	0.39	–0.005, 0.79	0.05	146	0.28	–0.13, 0.69	0.18
Depression score	148	–0.07	–0.39, 0.24	0.65	146	0.20	–0.12, 0.53	0.21	146	–0.01	–0.34, 0.32	0.94
Overall	148	–0.09	–0.60, 0.41	0.72	146	0.60	0.09, 1.11	0.02	146	0.27	–0.26, 0.79	0.32

Abbreviations: *β*, beta coefficient; BMI, body mass index; CI, confident interval; EHLI, Extended Healthy Lifestyle Index; FACIT, Functional Assessment of Chronic Illness Therapy; HADS, Hospital Anxiety and Depression Scale; HbA1c, glycated hemoglobin; MDS, Mediterranean diet score; WCRF/AICR, World Cancer Research Fund/American Institute of Cancer Research.

1 Adjusted for age (continuous), total energy intake (continuous), education level (high school or less / Technical and Further Education (TAFE) or diploma or university), marital status (partner/no partner), smoking (never / former / current), comorbidities (0 / 1+), physical activity (continuous, minutes/week), and BMI (only for MDS models that predict all outcomes except for BMI).

2 WCRF/AICR models were not adjusted for BMI or physical activity (incorporated in the score).

3 EHLI models were not adjusted for BMI or physical activity or smoking (incorporated in the score).

4 BMI outcome was not estimated for WCRF/AICR and EHLI (incorporated in the score).

In an exploratory subgroup analysis, the association between the WCRF/AICR score and overall anxiety and depression (HADS) score was more pronounced among younger patients (< 50 y), those with higher education levels, no history of smoking, lower BMI category (< 40 kg/m²), engagement in any physical activity, absence of comorbidities, and those without a partner, in multivariable models (Supplemental Table 7).

The scores of the WCRF/AICR and EHLI indices were highly correlated (*r =* 0.8, *P <* 0.0001) and showed moderate agreement (weighted kappa = 0.5, *P <* 0.0001). The MDS showed weaker correlations with both the WCRF/AICR (*r =* 0.2, *P* = 0.01) and the EHLI (*r =* 0.3, *P* = 0.001). Agreement between MDS and EHLI tertiles was fair (weighted kappa = 0.3, *P* < 0.0001), whereas agreement between MDS and WCRF/AICR was poor (weighted kappa = 0.1, *P* = 0.047), Supplemental Tables 8 and 9.

## Discussion

In 150 females enrolled in the FeMMe trial, mean diet and lifestyle quality scores were lower compared with the general population in Australia and other countries such as the United Kingdom and the United States, particularly in relation to adherence to the WCRF/AICR recommendations [[46], [47], [48]]. Most participants reported no physical activity, and their diets were primarily characterized by high intake of red and processed meat, refined grains, and sugary foods. Consistent with these lifestyle patterns, the mean waist circumference and waist-to-hip ratio measurements exceeded clinical guidelines. Additionally, higher quality diet and lifestyle patterns were associated with lower insulin resistance and better overall QoL, but also with higher anxiety and depression scores.

Our findings suggest that among females newly diagnosed with EC, despite having a BMI of 30 or more, a healthier lifestyle may be associated with lower fasting insulin levels and reduced insulin resistance, reinforcing the importance of diet and lifestyle quality. EC survivors with obesity, particularly abdominal obesity, and poor metabolic health have a higher risk of cardiometabolic comorbidities [5], which can be challenging to manage [49], complicate cancer treatment, and increase treatment-related complications [50]. Individuals with obesity who maintain normal insulin sensitivity have lower risk of cardiometabolic comorbidities, making glycemic control an important metabolic health goal [51]. Lifestyle and dietary factors play a significant role in modulating insulin levels and resistance [52]. Habitual physical activity is associated with lower insulin resistance, improved insulin sensitivity, and better glycemic control [53]. Furthermore, a diet rich in fiber and low in saturated fats and refined sugars, which is a characteristic of the dietary patterns recommended by the WCRF/AICR, can improve insulin sensitivity by modulating gut microbiota and reducing systemic inflammation [2]. According to the WCRF/AICR report, coffee is the only food component with convincing evidence supporting its role in EC prevention, likely through inflammation and insulin sensitivity pathways [2]. However, existing healthy dietary patterns often do not include coffee as a component. Other dietary patterns, such as the Empirical Dietary Inflammatory Pattern and the Empirical Dietary Index for Hyperinsulinemia, do incorporate coffee and have been associated with better outcomes in survivors of other cancer types [54,55]. In contrast, long-term consumption of a high glycemic load diet results in hyperinsulinemia, which in turn increases the bioavailability of insulin-like growth factor 1 and promotes EC [2]. Additionally, avoiding smoking and limiting alcohol consumption, particularly heavy drinking, which are also components of the EHLI, can prevent insulin resistance and the development of type 2 diabetes [56,57].

Our study suggests that adherence to the WCRF/AICR cancer prevention recommendations is associated with higher overall QoL in females with EC. Females in the FeMMe trial exhibited low QoL scores at baseline, particularly the emotional, functional, EC-specific, and general QoL domains. EC survivors frequently encounter a decline in their health-related QoL attributed to the cancer and related treatments [58]. Obesity and a sedentary lifestyle can exacerbate poor QoL [59], and contribute to decreased mobility, impaired cardiovascular health, and diminished psychological well-being after treatment. Given the low baseline QoL observed in this cohort, interventions to improve QoL during treatment should be considered such as lifestyle modifications, nutritional support, physical activity programs, psychological counseling, and social support. Only 12% of EC survivors follow the recommended lifestyle behaviors for cancer survivors as outlined by the American Cancer Society [60]. Several randomized controlled trials have demonstrated that healthy lifestyle interventions could enhance the QoL of EC survivors; however, engagement with these interventions has often been suboptimal [[60], [61], [62], [63]]. Studies on other predominantly female cancers such as breast cancer have also shown more promising results. For instance, females surviving breast cancer who followed the WCRF/AICR guideline experienced an improvement in their overall health status and QoL [64]. This includes higher scores for physical and role functioning and lower scores for symptoms such as fatigue, nausea, vomiting, pain, dyspnea, loss of appetite, and diarrhea.

Our study suggests a potential relationship between dietary and lifestyle factors and mental health outcomes in EC survivors. Unexpectedly, higher WCRF/AICR scores were associated with greater anxiety and depression, particularly among younger individuals, those with fewer comorbidities, and those without a partner. One possible explanation is that individuals experiencing psychological distress may adopt healthier diets and lifestyles, reflecting reverse causality. This contrasts with existing evidence, which generally links adverse mental health with poorer diet quality [65]. Alternatively, heightened health vigilance in certain subgroups may contribute to this pattern, with individuals more attuned to their health engaging in healthier behaviors despite psychological distress. Several methodological factors may also explain this unexpected association. Selection (collider) bias is a possibility, particularly given the characteristics of study participants. Collider bias arises when both the exposure and outcome influence a third variable that is conditioned upon, potentially distorting the observed associations [66]. Additionally, measurement error in self-reported diet, lifestyle behaviors, or psychological distress may have introduced bias or attenuated true associations, as such instruments are prone to inaccuracies due to recall limitations, social desirability, and variability in interpretation. Finally, the cross-sectional nature of the data limits our ability to establish temporality, making it difficult to determine the directionality of observed relationships and increasing susceptibility to reverse causation.

This study utilized three indices: MDS, WCRF/AICR score, and EHLI, which differ in scope and composition. The MDS focuses exclusively on dietary patterns, whereas the WCRF/AICR score and EHLI incorporate broader lifestyle factors such as physical activity, body composition, and alcohol intake. Including multiple constructs within a single index provides a more comprehensive view of health behaviors and may better reflect the multifactorial nature of cancer risk and survivorship. However, this approach can also limit interpretability, as it becomes challenging to disentangle the individual contributions of diet from other lifestyle behaviors. In our study, associations with metabolic health, QoL, and mental health varied across indices, suggesting that different constructs may influence outcomes in distinct ways. The use of both diet-specific and composite indices allowed for a more nuanced understanding of these relationships in females with EC.

Strengths of this study include detailed assessment of diet and lifestyle with validated tools, and comprehensive assessment of metabolic health, mental health, and QoL at baseline. We investigated dietary and lifestyle scores that are well-established and widely recognized in the field of nutritional epidemiology. Our study includes detailed information on cancer characteristics used to define the study population.

The study has several limitations that need to be considered when interpreting the results. Using cross-sectional analysis, the study captures data at a single point in time, which can identify correlations but not causality. The relatively small sample size may limit statistical power and the generalizability of findings. Measurement limitations related to self-reporting should be considered when interpreting results. Residual or unmeasured confounding (e.g., genetic predisposition) may also influence the results. Moreover, the study’s findings may not be generalizable to all females with EC, and dietary behaviors may change following diagnosis, highlighting the need for longitudinal research. Additionally, we used a modified version of the WCRF/AICR score, excluding salt intake, supplement use, and breastfeeding duration due to unavailable data, resulting in a total score range of 0–6. Although this captured key behaviors relevant to cancer prevention and survivorship, it limits comparability with studies using the full standardized score. Consistent implementation of the WCRF/AICR score is important for validity and comparability across studies [37].

Despite these limitations, our findings offer valuable insights into the associations between lifestyle factors and health outcomes in females with EC and can inform future research and intervention design.

In conclusions, a healthy lifestyle, including a high-quality diet and physical activity, was associated with metabolic health, QoL, and mental health among EC survivors. These findings may help inform the design of targeted lifestyle interventions aimed at improving outcomes in this population.

## Author contributions

The authors’ responsibilities were as follows – MJ, MP, RA: conceptualization, methodology, and writing—review and editing; RA: software, formal analysis, writing—original draft preparation, and visualization; MJ, MP, AO: validation, supervision; MJ, AO: resources and funding acquisition; MJ, AO, RA: data curation; AO: project administration; and all authors: read and approved the final manuscript.

## Data and code availability

Raw data access is restricted for patient confidentiality. All analysis code used in this study is available at: https://github.com/RuqaiyaAR/FeMMe-analysis-code/blob/main/FeMMe%20baseline%20diet%20analysis%20codes.sas.

## Declaration of AI and AI-Assisted Technologies in the Writing Process

During the preparation of this work, the author(s) utilized “Copilot AI” to enhance the SAS code used for analysis and to refine the language. After using this tool, the author(s) reviewed and edited the content as needed and take(s) full responsibility for the content of the publication.

## Funding

This research received no external funding. The FeMMe trial, however, was funded by Cancer Australia, UQ academic Title Holders Grant, Royal Brisbane and Women’s Hospital Foundation, Lord Mayor’s Community Fund, and Australian and New Zealand Gynaecologic Oncology Group.

## Conflict of interest

The authors report no conflicts of interest.
