# Supplementary material for: Diet and Lifestyle Quality in Australian Females with Endometrial Cancer at Diagnosis: Insights from the Fertility-sparing Management for Early Endometrial Cancer (FeMMe) Trial
Source: Curr Dev Nutr. 2025 Oct 11;9(12):107572. doi: 10.1016/j.cdnut.2025.107572 (PMC12718200; doi:10.1016/j.cdnut.2025.107572)
Supplement: Multimedia component 1 [file mmc1.docx]

Supplementary Material for "Diet and lifestyle quality in Australian women with endometrial cancer at diagnosis – insights from the FeMMe trial" – First Author: Ruqaiya Al Ramadhani

FeMMe participants at baseline
N=165

Excluded
Women with missing dietary data at baseline (N=8)

Missing baseline demographic (N=1)

N=156

Excluded:

Women with implausible energy intake at baseline (N=6)

N=150

Included in the diet-only analysis

Excluded:

Women with unknown level of physical activity (N=3)

N=147

Included in the diet and lifestyle analysis

## Supplementary Figure 1: Flowchart of study participants

## Supplementary Table S1: Dietary recommendations included in the Mediterranean Diet Score (MDS) calculation.

| Food group | Food included | Criteria for 1 point |
| --- | --- | --- |
| Vegetables | All vegetables (excluding potatoes, chips, and tomato sauce) (g/d) | ≥Median intake |
| Fruit and nuts | All fruits (excluding fruit juices, nuts, and peanut butter) (g/d) | ≥Median intake |
| Legumes | All beans, tofu (g/d) | ≥Median intake |
| Cereals | Wholemeal grains, rice (g/d) | ≥Median intake |
| Fish | All Fish (g/d) | ≥Median intake |
| Milk and dairy products | All Milk, cheese, and yogurt (g/d) | ≤Median intake |
| Red and processed meats | beef, lamb, pork, veal, hamburger, sausage, bacon, salami, meat pie, ham (g/d) | ≤Median intake |
| Monounsaturated to saturated fat ratio | Ratio of monounsaturated fat (g) to saturated fat (g) | ≥Median intake |
| Ethanol | Heavy beer, light beer, red wine, white wine, fortified wines, spirits (g/d) | 5- 25g |

Abbreviations: g = gram, g/d = grams per day.

## Supplementary Table S2: Dietary and lifestyle recommendations included the World Cancer Research Fund / American Institute for Cancer Research (WCRF/AICR) score calculation.

| Recommendations | Diet/ lifestyle items included in the calculation | Criteria for 1 point |
| --- | --- | --- |
| BMI | BMI kg/m^2^ categories (All obese category) | BMI 18.5–24.9 kg/m^2^ |
| Physical activity | Moderate or vigorous activity (min/day) – Vigorous activity, double-weighted relative to moderate activity for scoring purposes | ≥ 30 min/day of moderate activity, or ≥ 15 min/day vigorous activity |
| Foods and drinks that promote weight gain | - Energy density (ED) was calculated as energy (kcal) from all foods and drinks divided by their weight in grams (g).    - Sugary drinks: fruit juice + soft drinks (g/d) | Energy density **≤**125 kcal/100g  Sugary drinks < 250g/day |
| Plant foods | - Fruit and vegetables: Apple, pear, orange, banana, tinned fruit, apricots, peaches, strawberries, melon, mango, pineapple, avocado, pumpkin, carrots, tomatoes, cabbage, capsicum, cauliflower, mushrooms, onion, garlic, zucchini, cucumber, celery, beetroot broccoli, bean sprouts, spinach, lettuce, peas, green beans, baked beans, other beans, Tofu (excluding chips, tomato sauce, and potatoes) (g/d)    - Total fiber intake: calculated from dietary fiber (nutrient) (g/d)    - Refined grains: white bread, high fiber white bread, cornflakes, rice, pasta, crackers, crisps, cakes, sweet biscuits, meat pie, pizza, rice (type not specified in FFQ) (g/d) | Fruit and vegetables ≥ 400 g/d  Fiber intake ≥ 25 g/d  Tertile 1 (no cut-offs in the guideline) |
| Animal foods | - Red meat: beef, lamb, pork, veal, hamburger (g/week)  - Processed meat: sausage, bacon, salami, meat pie, ham (g/week) | Red meat < 500 g/week and processed meat < 50 g/week |
| Alcoholic drinks | Heavy beer, light beer, red wine, white wine, fortified wines, spirits (g/d) | Alcohol ≤ 10 g/d |
| Salt | Limit intake, Australian cut-off salt intake (use NA <= 1.6 g is low risk, =>2.3 is high risk) – Not included (no data) | Not included |
| Dietary supplements | Supplements, Yes/No – Not included (no data) | Not included |
| Breastfeeding | Cumulative breastfeeding recommendations: >=6 months – Not included (no data on duration, only yes/no data available) | Not included |

Abbreviations: BMI = Body Mass Index, ED = Energy Density, g = gram; g/d = grams per day, g/week = grams per week, min/day = minutes per day, kcal = kilocalories, kg/m² = kilograms per square meter, WCRF/AICR = World Cancer Research Fund / American Institute for Cancer Research.

## Supplementary Table S3: Dietary and lifestyle recommendations included in the Extended Healthy Lifestyle Index (EHLI) score calculation.

| Recommendations | Diet/lifestyle items included in the calculation | Criteria for 1 point |
| --- | --- | --- |
| Smoking | Never, former, current | Never smoker |
| BMI | BMI kg/m^2^ categories (All obese category) | BMI 18.5–24.9 kg/m^2^ |
| Physical activity | Moderate or vigorous activity (min/week) – vigorous activity double-weighted relative to moderate activity for scoring purposes | ≥300 min/week of moderate activity, or  ≥150 min/week of vigorous activity |
| Dietary pattern that is high in vegetables, fruits, and whole grains, less in drinks that promote weight gain red and processed meat | - Red and processed meat: beef, lamb, pork, veal, hamburger, sausage, bacon, salami, meat pie, ham (g/d)  - Drinks that promote weight gain: fruit juice + soft drinks (g/d)    - Fruit and vegetables: Apple, pear, orange, banana, tinned fruit, apricots, peaches, strawberries, melon, mango, pineapple, avocado, pumpkin, carrots, tomatoes, cabbage, capsicum, cauliflower, mushrooms, onion, garlic, zucchini, cucumber, celery, beetroot broccoli, spinach, lettuce (excluding chips, tomato sauce, and potatoes) (g/d)    - Unprocessed grains and legumes: oatmeal porridge, wholemeal bread, rye bread, multi-grain bread, all bran, bran flakes, weet-bix, muesli, corn flakes, rice, peas, green beans, bean sprouts, baked beans, other beans, tofu (g/d)  - Dairy products: All milk, cheese, and yogurt (g/d) | Red and processed meat < 23.23 g/d  Sugary drinks < 76.33 g/d  Fruit and vegetables ≥ 400 g/d  Unprocessed grains and legumes ≥ 295.33 g/d  Dairy ≥ 206 g/d |
| Alcohol | Heavy beer, light beer, red wine, white wine, fortified wines, spirits (g/d) | Alcohol <10 g/d |

Abbreviations: BMI = Body Mass Index, g = gram, g/d = grams per day, min/week = minutes per week, EHLI = Extended Healthy Lifestyle Index.

## Supplementary Table S4: Baseline characteristics of FeMMe participants by tertiles of the Mediterranean Diet Score (MDS) (N=150).

| **Baseline characteristics** | **N = 150**  **N (%)** | **MDS score** | | | **p-value** |
| --- | --- | --- | --- | --- | --- |
|  |  | **Tertile 1 (Min = 0, Max = 3.0)**  **N = 49**  **N (%)** | **Tertile 2**  **(Min =4.0, Max= 4.0)**  **N = 40**  **N (%)** | **Tertile 3**  **(Min = 5.0, Max = 8.0)**  **N =6 1**  **N (%)** |  |
| **Age in years, median (IQR)** | 150 | 54.1 (24.6) | 56.8 (23.0) | 56.6 (13.8) | 0.38 |
| **Race** |  |  |  |  |  |
| White | 103 (68.7) | 40 (81.6) | 24 (60.0) | 39 (63.9) | 0.05 |
| Non white/others | 47 (31.3) | 9 (18.4) | 16 (40.0) | 22 (36.1) |  |
| **Education level** |  |  |  |  |  |
| High school or less | 80 (53.7) | 29 (59.2) | 19 (47.5) | 32 (53.3) | 0.54 |
| TAFE or diploma, or University | 69 (46.3) | 20 (40.8) | 21 (52.5) | 28 (46.7) |  |
| Missing | 1 | 0 | 0 | 1 |  |
| **Income (annual)** |  |  |  |  |  |
| Low (<$10,000 – $20,000) | 35 (23.3) | 10 (21.3) | 11 (28.2) | 14 (24.6) | 0.27 |
| Middle (>$20,000 – $60,000) | 57 (38.0) | 23 (48.9) | 10 (25.6) | 24 (24.6) |  |
| High (>$60,000) | 51 (34.0) | 14 (29.8) | 18 (46.2) | 19 (33.3) |  |
| Missing | 7 | 2 | 1 | 4 |  |
| **Marital status** |  |  |  |  |  |
| Partner | 88 (59.1) | 27 (56.3) | 21 (52.5) | 40 (65.6) | 0.38 |
| No partner | 61 (40.9) | 21 (43.8) | 19 (47.5) | 21 (34.4) |  |
| Missing | 1 | 1 | 0 | 0 |  |
| **Menopausal status** |  |  |  |  |  |
| Pre | 61 (40.7) | 24 (49.0) | 15 (37.5) | 22 (36.1) | 0.35 |
| Post | 89 (59.3) | 25 (51.0) | 25 (62.5) | 39 (63.9) |  |
| **Smoking** |  |  |  |  |  |
| Never | 82 (54.7) | 29 (59.2) | 24 (60.0) | 29 (47.5) | 0.67** |
| Former | 54 (36.0) | 16 (30.6) | 16 (32.5) | 26 (42.6) |  |
| Current | 14 (9.3) | 5 (10.2) | 3 (7.5) | 6 (9.8) |  |
| **Family History of cancer** |  |  |  |  |  |
| No | 33 (24.6) | 8 (19.5) | 8 (22.2) | 17 (29.8) | 0.47 |
| Yes | 101 (75.4) | 33 (80.5) | 28 (77.8) | 40 (70.2) |  |
| Missing | 16 | 8 | 4 | 4 |  |
| **Diabetes** |  |  |  |  |  |
| No | 104 (69.3) | 36 (73.5) | 30 (75.0) | 38 (62.3) | 0.30 |
| Yes | 46 (30.7) | 13 (26.5) | 10 (25.0) | 23 (37.7) |  |
| **Depression** |  |  |  |  |  |
| No | 128 (85.3) | 44 (89.9) | 32 (80.0) | 52 (85.3) | 0.43 |
| Yes | 22 (14.7) | 5 (10.2) | 8 (20.0) | 9 (14.8) |  |
| **Anxiety** |  |  |  |  |  |
| No | 138 (92.0) | 45 (91.8) | 36 (90.0) | 57 (93.4) | 0.87** |
| Yes | 12 (8.0) | 4 (8.2) | 4 (10.0) | 4 (6.7) |  |
| **Comorbidities^a^** |  |  |  |  |  |
| 0 | 52 (34.7) | 16 (32.7) | 17 (42.5) | 19 (31.2) | 0.47 |
| 1+ | 98 (65.3) | 33 (67.4) | 23 (57.5) | 42 (68.9) |  |
| **Physical activity** |  |  |  |  |  |
| No | 114 (77.6) | 38 (79.2) | 30 (75.0) | 46 (78.0) | 0.89 |
| Yes | 33 (22.5) | 10 (20.8) | 10 (25.0) | 13 (22.0) |  |
| Missing | 3 | 1 | 0 | 2 |  |
| **Glycaemic control parameters** |  |  |  |  |  |
| HbA1c (%), median (IQR) | 149 | 5.8 (1.1) | 5.6 (0.7) | 6.0 (1.0) | 0.32 |
| Fasting insulin (nmol/L), median (IQR) | 135 | 21.5 (22.5) | 20.5 (19.2) | 17.0 (14.0) | 0.25 |
| Fasting glucose (mmol/L), median (IQR) | 141 | 5.9 (1.8) | 5.6 (1.3) | 6.2 (2.4) | 0.31 |
| HOMA- IR, median (IQR) | 133 | 6.0 (6.0) | 4.8 (6.5) | 5.6 (5.1) | 0.39 |
| **Body composition** |  |  |  |  |  |
| BMI (Kg/m^2^), mean (SD) | 150 | 47.9 (9.5) | 47.0 (9.9) | 47.4 (8.9) | 0.91 |
| Waist (cm), mean (SD) | 142 | 129.1 (16.9) | 127.6 (18.5) | 131.6 (16.0) | 0.51 |
| Waist/Hip ratio, mean (SD) | 142 | 0.9 (0.1) | 0.9 (0.1) | 0.9 (0.1) | 0.56 |
| **Quality of life (FACIT score)** |  |  |  |  |  |
| Physical, mean (SD) | 148 | 20.9 (5.9) | 21.3 (4.9) | 20.1 (5.6) | 0.57 |
| Social, mean (SD) | 147 | 18.7 (6.8) | 20.2 (6.3) | 18.2 (6.1) | 0.32 |
| Emotional, mean (SD) | 147 | 17.2 (4.5) | 18.3 (4.4) | 15.8 (4.4) | **0.03** |
| Functional, mean (SD) | 148 | 18.8 (7.0) | 20.4 (5.3) | 16.9 (6.0) | **0.02** |
| Additional, mean (SD) | 148 | 47.0 (13.2) | 49.9 (8.1) | 47.9 (8.9) | 0.42 |
| General, mean (SD) | 148 | 122.2 (29.8) | 130.0 (20.1) | 118.6 (21.7) | 0.08 |
| **Depression and Anxiety scores (HADS)** |  |  |  |  |  |
| Anxiety score, mean (SD) | 148 | 13.0 (3.2) | 13.6 (2.8) | 12.2 (2.9) | 0.06 |
| Depression score, mean (SD) | 148 | 14.5 (2.3) | 15.2 (2.1) | 14.5 (2.2) | 0.17 |
| Overall, mean (SD) | 148 | 27.5 (4.9) | 28.9 (4.5) | 26.6 (4.2) | 0.06 |

Abbreviations: MDS = Mediterranean Diet Score, SD=standard deviation, IQR = Interquartile range, HbA1c = glycated haemoglobin, HOMA- IR= Homeostasis Model Assessment – Insulin Resistance, BMI = Body mass index, cm = centimetres, kg/m² = kilograms per square metre, mmol/L = millimoles per litre, nmol/L = nanomoles per litre, FACIT = Functional Assessment of Chronic Illness Therapy, HADS = The Hospital Anxiety and Depression Scale.

*p-trend

**Fisher’s exact p-value

^a^ Based on the Charlson Comorbidity Index score

## Supplementary Table S5: Baseline characteristics of FeMMe participants by tertiles of the World Cancer Research Fund/American Institute of Cancer Research (WCRF/AICR) score (N=147).

| **Baseline characteristics** | **N = 147**  **N (%)** | **WCRF/AICR score** | | | **p-value** |
| --- | --- | --- | --- | --- | --- |
|  |  | **Tertile 1 (Min = 0.17, Max = 1.67)**  **N = 46**  **N (%)** | **Tertile 2 (Min = 1.75, Max=2.25)**  **N = 55**  **N (%)** | **Tertile 3 (Min = 2.33, Max = 4.33)**  **N = 46**  **N (%)** |  |
| **Age in years, median (IQR)** | 147 | 54.5 (24.1) | 54.2 (21.9) | 61.5 (13.6) | **0.001** |
| **Race** |  |  |  |  |  |
| White | 101 (68.7) | 37 (80.4) | 37 (67.3) | 27 (58.7) | 0.08 |
| Non white/others | 46 (31.3) | 10 (19.6) | 18 (32.7) | 19 (41.3) |  |
| **Education level** |  |  |  |  |  |
| High school or less | 78 (53.1) | 23 (50.0) | 21 (38.2) | 34 (73.9) | **0.02*** |
| TAFE or diploma, or University | 69 (46.9) | 23 (50.0) | 34 (61.8) | 12 (26.1) |  |
| **Income (annual)** |  |  |  |  |  |
| Low (<$10,000 – $20,000) | 35 (24.7) | 12 (26.1) | 8 (15.4) | 15 (34.1) | 0.13 |
| Middle (>$20,000 – $60,000) | 56 (39.4) | 14 (30.4) | 25 (48.1) | 17 (38.6) |  |
| High (>$60,000) | 51 (35.9) | 20 (43.5) | 19 (36.5) | 12 (27.3) |  |
| Missing | 5 | 0 | 3 | 5 |  |
| **Marital status** |  |  |  |  |  |
| Partner | 87 (59.6) | 29 (63.0) | 31 (56.4) | 27 (60.0) | 0.79 |
| No partner | 59 (40.4) | 17 (37.0) | 24 (43.6) | 18 (40.0) |  |
| Missing | 1 | 0 | 0 | 1 |  |
| **Menopausal status** |  |  |  |  |  |
| Pre | 59 (40.1) | 20 (43.5) | 28 (50.9) | 11 (23.9) | **0.02** |
| Post | 88 (59.9) | 26 (56.5) | 27 (49.1) | 35 (76.1) |  |
| **Smoking** |  |  |  |  |  |
| Never | 82 (55.8) | 24 (52.2) | 34 (61.8) | 24 (52.2) | 0.42** |
| Former | 52 (35.4) | 16 (34.8) | 16 (29.1) | 20 (43.5) |  |
| Current | 13 (8.8) | 6 (13.0) | 5 (9.1) | 2 (4.4) |  |
| **Family History of Cancer** |  |  |  |  |  |
| No | 33 (25.2) | 13 (32.5) | 12 (25.0) | 8 (18.6) | 0.35 |
| Yes | 98 (74.8) | 27 (67.5) | 36 (75.0) | 35 (81.4) |  |
| Missing | 16 | 6 | 7 | 3 |  |
| **Diabetes** |  |  |  |  |  |
| No | 102 (69.4) | 30 (65.2) | 42 (76.4) | 30 (65.2) | 0.37 |
| Yes | 45 (30.6) | 16 (34.8) | 13 (23.6) | 16 (34.8) |  |
| **Depression** |  |  |  |  |  |
| No | 127 (86.4) | 41 (89.1) | 50 (90.9) | 36 (78.3) | 0.15 |
| Yes | 20 (13.6) | 5 (10.9) | 5 (9.1) | 10 (21.7) |  |
| **Anxiety** |  |  |  |  |  |
| No | 135 (91.8) | 44 (93.5) | 52 (94.6) | 40 (87.0) | 0.41** |
| Yes | 12 (8.2) | 3 (6.5) | 3 (5.5) | 6 (13.0) |  |
| **Comorbidities^a^** |  |  |  |  |  |
| 0 | 51 (34.7) | 18 (39.1) | 23 (41.8) | 10 (21.7) | 0.08 |
| 1+ | 96 (65.3) | 28 (60.9) | 32 (58.2) | 36 (78.3) |  |
| **Physical activity** |  |  |  |  |  |
| No | 114 (77.6) | 36 (78.3) | 44 (80.0) | 34 (73.9) | 0.76 |
| Yes | 33 (22.5) | 10 (21.7) | 11 (20.0) | 12 (26.1) |  |
| **Glycaemic control parameters** |  |  |  |  |  |
| HbA1c (%), median (IQR) | 146 | 5.9 (0.9) | 5.7 (1.4) | 5.8 (1.1) | 0.48 |
| Fasting insulin (nmol/L), median (IQR) | 132 | 22.6 (20.0) | 19.0 (17.0) | 17.0 (15.0) | **0.04** |
| Fasting glucose (mmol/L), median (IQR) | 138 | 5.7 (1.3) | 5.8 (2.0) | 6.0 (2.2) | 0.42 |
| HOMA- IR, median (IQR) | 130 | 6.1 (5.5) | 4.7 (7.3) | 4.6 (3.8) | 0.09 |
| **Body composition** |  |  |  |  |  |
| BMI (Kg/m^2^), mean (SD) | 147 | 46.8 (9.0) | 47.8 (10.0) | 47.5 (8.8) | 0.85 |
| Waist (cm), mean (SD) | 139 | 129.5 (16.3) | 130.1 (16.5) | 129.5 (18.7) | 1.00 |
| Waist/Hip ratio, mean (SD) | 139 | 0.9 (0.1) | 0.9 (0.1) | 0.9 (0.1) | 0.74 |
| **Quality of life (FACIT score)** |  |  |  |  |  |
| Physical, mean (SD) | 146 | 20.8 (6.2) | 20.5 (4.3) | 20.9 (5.7) | 0.91 |
| Social, mean (SD) | 145 | 18.4 (6.7) | 19.7 (6.0) | 19.1 (6.0) | 0.58 |
| Emotional, mean (SD) | 145 | 15.8 (4.9) | 17.0 (4.3) | 18.0 (4.3) | 0.07 |
| Functional, mean (SD) | 146 | 17.7 (6.7) | 18.9 (5.6) | 19.0 (6.5) | 0.52 |
| Additional, mean (SD) | 146 | 46.9 (12.1) | 47.9 (9.5) | 50.0 (9.4) | 0.35 |
| General, mean (SD) | 146 | 118.7 (27.4) | 123.9 (21.6) | 127.0 (24.3) | 0.27 |
| **Depression and Anxiety scores (HADS)** |  |  |  |  |  |
| Anxiety score, mean (SD) | 146 | 12.2 (3.1) | 12.9 (3.3) | 13.5 (2.4) | 0.13 |
| Depression score, mean (SD) | 146 | 14.2 (2.2) | 14.7 (2.2) | 15.2 (2.1) | 0.12 |
| Overall, mean (SD) | 146 | 26.4 (4.8) | 27.6 (4.7) | 28.6 (4.0) | 0.07 |

Abbreviations: WCRF/AICR = World Cancer Research Fund/American Institute of Cancer Research, SD = standard deviation, IQR = Interquartile range, HbA1c = glycated haemoglobin, HOMA- IR = Homeostasis Model Assessment – Insulin Resistance, BMI = Body mass index, cm = centimetres, kg/m² = kilograms per square metre, mmol/L = millimoles per litre, nmol/L = nanomoles per litre, FACIT=Functional Assessment of Chronic Illness Therapy, HADS = The Hospital Anxiety and Depression Scale.

*p-trend

**Fisher’s exact p-value

^a^ Based on the Charlson Comorbidity Index score

## Supplementary Table S6: Baseline characteristics of FeMMe participants by tertiles of the Extended Healthy Lifestyle Index (EHLI) score (N=147).

| **Baseline characteristics** | **N = 147**  **N (%)** | **EHLI score** | | | **p-value** |
| --- | --- | --- | --- | --- | --- |
|  |  | **Tertile 1 (Min = 1.0, Max = 3.0)**  **N = 56**  **N (%)** | **Tertile 2 (Min = 3.5, Max = 4.0)**  **N = 40**  **N (%)** | **Tertile 3 (Min = 4.5, Max = 7.0)**  **N = 51**  **N (%)** |  |
| **Age in years, median (IQR)** | 147 | 54.5 (24.8) | 56.8 (19.7) | 57.1 (11.8) | 0.19 |
| **Race** |  |  |  |  |  |
| White | 101 (68.7) | 42 (75.0) | 32 (60.0) | 35 (68.6) | 0.29 |
| Non white/others | 46 (31.3) | 14 (25.0) | 16 (40.0) | 16 (31.4) |  |
| **Education level** |  |  |  |  |  |
| High school or less | 78 (53.1) | 25 (44.6) | 24 (60.0) | 29 (56.9) | 0.20* |
| TAFE or diploma, or University | 69 (46.9) | 31 (55.4) | 16 (40.0) | 22 (43.1) |  |
| **Income (annual)** |  |  |  |  |  |
| Low (<$10,000 – $20,000) | 35 (24.7) | 14 (25.5) | 6 (16.2) | 15 (30.0) | 0.53 |
| Middle (>$20,000 – $60,000) | 56 (39.4) | 19 (34.6) | 17 (46.0) | 20 (40.0) |  |
| High (>$60,000) | 51 (35.9) | 22 (40.0) | 14 (37.8) | 15 (30.0) |  |
| Missing | 5 | 1 | 3 | 1 |  |
| **Marital status** |  |  |  |  |  |
| Partner | 87 (59.6) | 33 (58.9) | 23 (59.0) | 31 (60.8) | 0.98 |
| No partner | 59 (40.4) | 23 (41.1) | 16 (41.0) | 20 (39.2) |  |
| Missing | 1 | 0 | 1 | 0 |  |
| **Menopausal status** |  |  |  |  |  |
| Pre | 59 (40.1) | 25 (44.6) | 17 (42.5) | 17 (33.3) | 0.46 |
| Post | 88 (59.9) | 31 (55.4) | 23 (57.5) | 35 (66.7) |  |
| **Smoking** |  |  |  |  |  |
| Never | 82 (55.8) | 27 (48.2) | 21 (52.5) | 34 (66.7) | **0.02**** |
| Former | 52 (35.4) | 20 (35.7) | 15 (37.5) | 17 (33.3) |  |
| Current | 13 (8.8) | 9 (16.1) | 4 (10.0) | 0 (0.0) |  |
| **Family History of Cancer** |  |  |  |  |  |
| No | 33 (25.2) | 17 (34.7) | 7 (20.0) | 9 (19.2) | 0.15 |
| Yes | 98 (74.8) | 32 (65.3) | 28 (80.0) | 38 (80.9) |  |
| Missing | 16 | 7 | 5 | 4 |  |
| **Diabetes** |  |  |  |  |  |
| No | 102 (69.4) | 38 (67.9) | 28 (70.0) | 36 (70.6) | 0.95 |
| Yes | 45 (30.6) | 18 (32.1) | 12 (30.0) | 15 (29.4) |  |
| **Depression** |  |  |  |  |  |
| No | 127 (86.4) | 50 (89.3) | 38 (95.0) | 39 (76.5) | **0.03** |
| Yes | 20 (13.6) | 6 (10.7) | 2 (5.0) | 12 (23.5) |  |
| **Anxiety** |  |  |  |  |  |
| No | 135 (91.8) | 53 (94.6) | 37 (92.5) | 45 (88.2) | 0.47** |
| Yes | 12 (8.2) | 3 (5.4) | 3 (7.5) | 6 (11.8) |  |
| **Comorbidities^a^** |  |  |  |  |  |
| 0 | 51 (34.7) | 24 (42.9) | 12 (30.0) | 15 (29.4) | 0.26 |
| 1+ | 96 (65.3) | 32 (57.1) | 28 (70.0) | 36 (70.6) |  |
| **Physical activity** |  |  |  |  |  |
| No | 114 (77.6) | 43 (76.8) | 31 (77.5) | 40 (78.4) | 0.98 |
| Yes | 33 (22.5) | 13 (23.2) | 9 (22.5) | 11 (21.6) |  |
| **Glycaemic control parameters** |  |  |  |  |  |
| HbA1c (%), median (IQR) | 146 | 5.8 (1.0) | 5.7 (1.7) | 5.7 (0.8) | 0.12 |
| Fasting insulin (nmol/L), median (IQR) | 132 | 20.6 (17.6) | 21.0 (22.0) | 16.5 (14.0) | 0.06 |
| Fasting glucose (mmol/L), median (IQR) | 138 | 5.8 (2.2) | 5.8 (2.5) | 6.0 (1.4) | 0.08 |
| HOMA- IR, median (IQR) | 130 | 5.6 (4.5) | 6.1 (6.4) | 4.7 (3.6) | 0.07 |
| **Body composition** |  |  |  |  |  |
| BMI (Kg/m^2^), mean (SD) | 147 | 45.9 (8.1) | 48.9 (10.7) | 47.8 (9.2) | 0.29 |
| Waist (cm), mean (SD) | 139 | 129.1 (15.5) | 132.2 (17.9) | 128.3 (18.0) | 0.53 |
| Waist/Hip ratio, mean (SD) | 139 | 0.9 (0.1) | 0.9 (0.1) | 0.9 (0.1) | 0.15 |
| **Quality of life (FACIT score)** |  |  |  |  |  |
| Physical, mean (SD) | 146 | 20.0 (6.0) | 21.7 (5.3) | 20.8 (5.2) | 0.32 |
| Social, mean (SD) | 145 | 19.0 (6.2) | 20.4 (6.1) | 18.3 (6.2) | 0.28 |
| Emotional, mean (SD) | 145 | 16.1 (4.8) | 17.9 (4.7) | 17.0 (4.0) | 0.15 |
| Functional, mean (SD) | 146 | 17.3 (6.4) | 20.9 (5.3) | 18.1 (6.4) | **0.02** |
| Additional, mean (SD) | 146 | 47.3 (11.8) | 48.7 (10.0) | 48.9 (9.0) | 0.69 |
| General, mean (SD) | 146 | 119.0 (25.9) | 129.5 (24.3) | 123.1 (22.2) | 0.12 |
| **Depression and Anxiety scores (HADS)** |  |  |  |  |  |
| Anxiety score, mean (SD) | 146 | 12.4 (3.1) | 13.2 (3.0) | 13.0 (2.9) | 0.34 |
| Depression score, mean (SD) | 146 | 14.3 (2.4) | 15.0 (1.) | 14.9 (2.2) | 0.22 |
| Overall, mean (SD) | 146 | 26.7 (5.1) | 28.2 (4.2) | 28.0 (4.2) | 0.20 |

Abbreviations: EHLI = Extended Healthy Lifestyle Index, SD = standard deviation, IQR = Interquartile range, HbA1c = glycated haemoglobin, HOMA- IR = Homeostasis Model Assessment – Insulin Resistance, BMI=Body mass index, cm = centimetres, kg/m² = kilograms per square metre, mmol/L = millimoles per litre, nmol/L = nanomoles per litre, FACIT = Functional Assessment of Chronic Illness Therapy, HADS = The Hospital Anxiety and Depression Scale.

*p-trend

**Fisher’s exact p-value

^a^ Based on the Charlson Comorbidity Index score

(a)


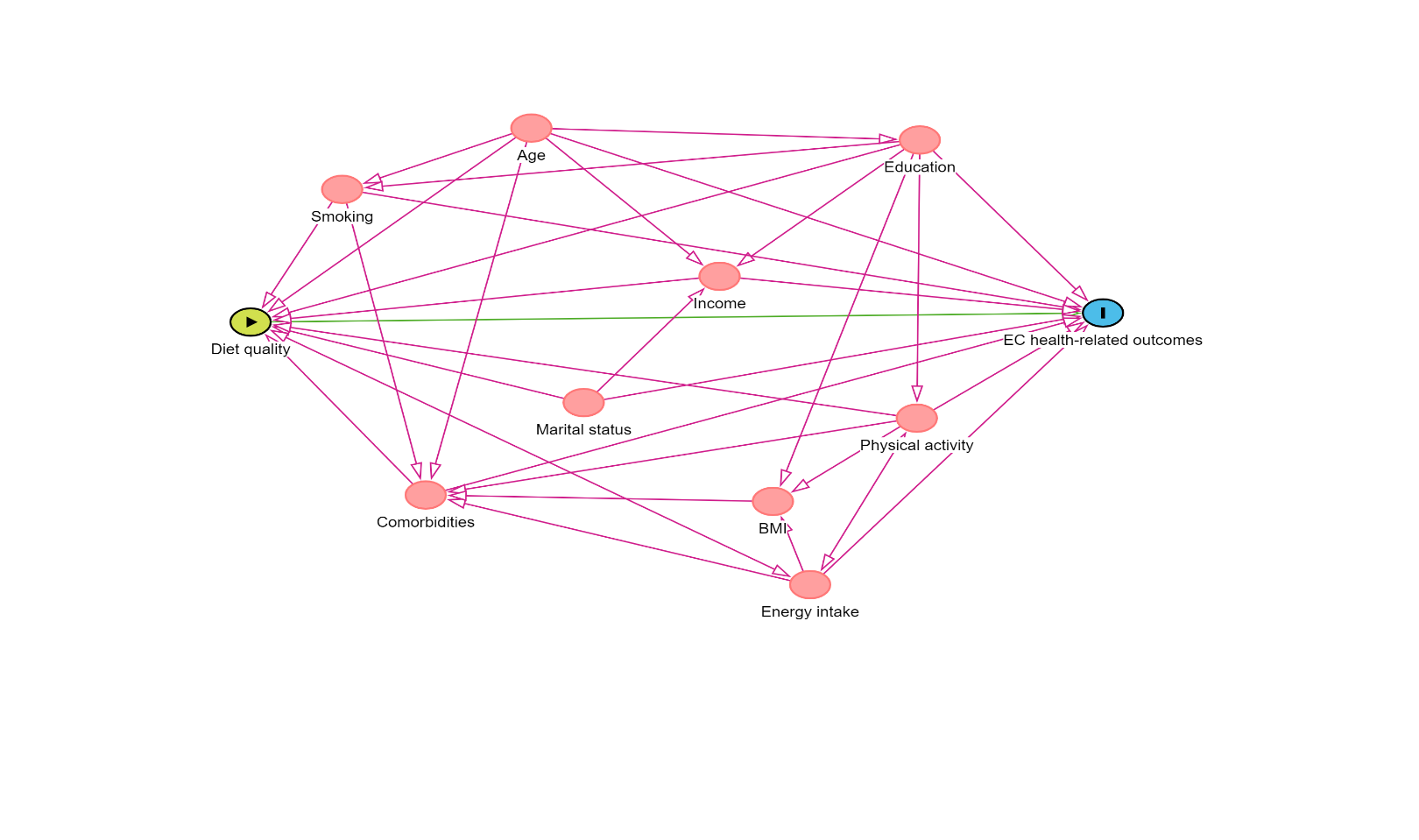

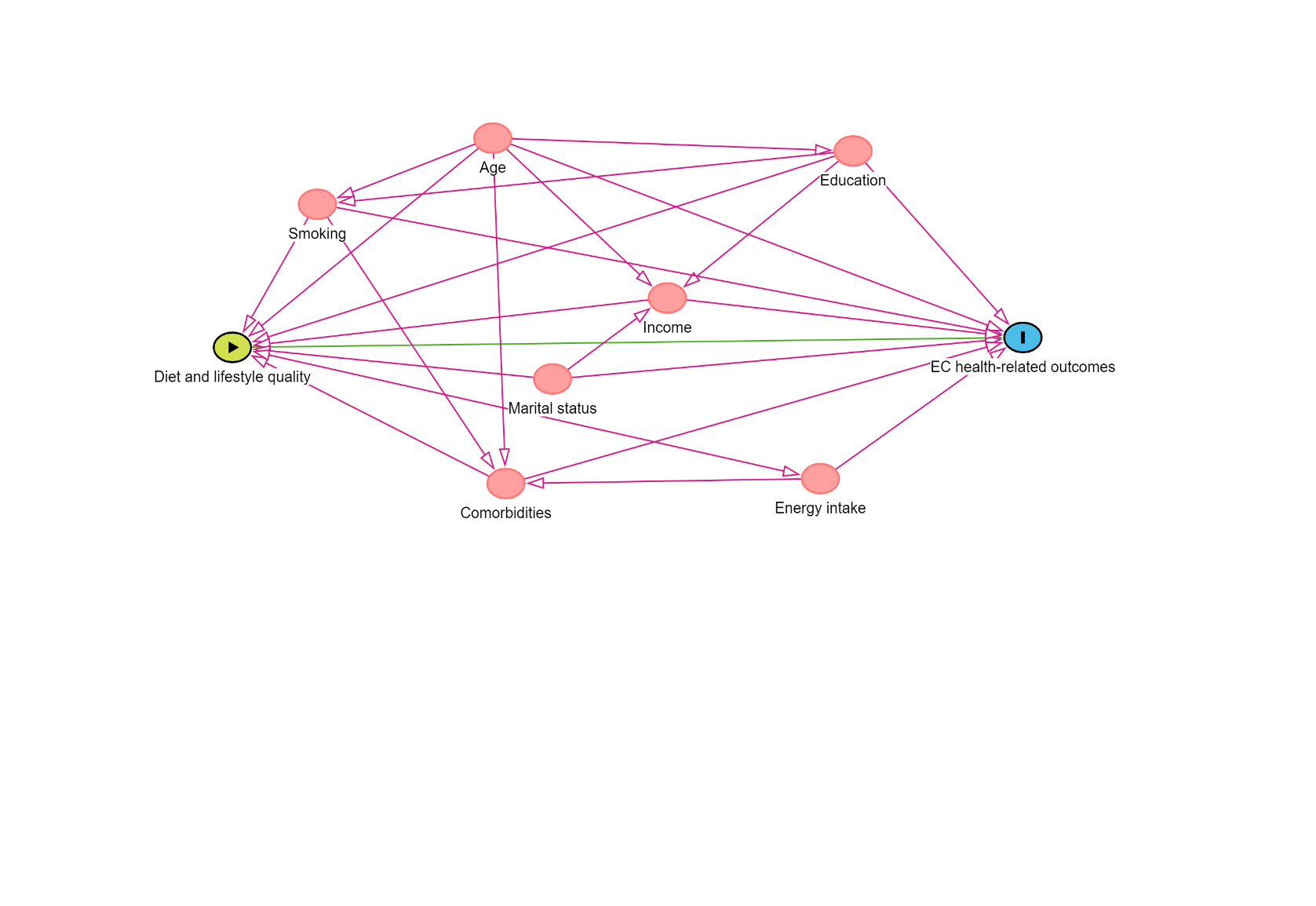


(b)

## Supplementary Figure 2: Directed Acyclic Graphs of causal relationship between diet quality (a) and diet and lifestyle quality (b) and EC health-related outcomes.

Abbreviations: BMI = Body Mass Index, EC = Endometrial Cancer

| **Baseline characteristics** | **N = 147**  **N (%)** | **WCRF/AICR score** | **p-value** |
| --- | --- | --- | --- |
|  |  | **Beta Coeff* (95% CI)** |  |
| **Age category** |  |  |  |
| <50 | 50 (34.0) | 2.63 (1.36 – 3.89) | **0.0001** |
| 50 - 65 | 64 (43.5) | 0.18 (-0.73 – 1.09) | 0.70 |
| >65 | 33 (22.5) | -0.25 (-2.31 – 1.80) | 0.80 |
| **Education level** |  |  |  |
| High school or less | 78 (53.1) | 0.71 (-0.20 – 1.61) | 0.12 |
| TAFE or diploma, or University | 69 (46.9) | 1.26 (0.09 – 2.42) | **0.04** |
| **Income (annual)** |  |  |  |
| Low (<$10,000 – $20,000) | 35 (24.7) | -0.88 (-2.81 – 1.04) | 0.35 |
| Middle (>$20,000 – $60,000) | 56 (39.4) | 0.81 (-0.38 – 2.01) | 0.18 |
| High (>$60,000) | 51 (35.9) | 0.94 (-0.26 – 2.14) | 0.12 |
| Missing | 5 | - |  |
| **Marital status** |  |  |  |
| Partner | 87 (59.6) | 0.42 (-0.87 – 1.71) | 0.52 |
| No partner | 59 (40.4) | 0.91 (0.10 – 1.72) | **0.03** |
| Missing | 1 | - |  |
| **Smoking** |  |  |  |
| Never | 82 (55.8) | 1.20 (0.27 – 2.13) | **0.01** |
| Former | 52 (35.4) | -0.10 (-1.34 – 1.14) | 0.87 |
| Current | 13 (8.8) | 1.70 (-3.27 – 6.68) | 0.43 |
| **BMI category** |  |  |  |
| <40 Kg/m^2^ | 30 (20.4) | 1.80 (0.39 – 3.22) | **0.01** |
| 40-50 Kg/m^2^ | 69 (46.9) | 0.01 (-1.09 – 1.11) | 0.98 |
| >50 Kg/m^2^ | 48 (32.7) | 1.05 (-0.40 – 2.50) | 0.15 |
| **Reported anxiety and/or depression** |  |  |  |
| No | 121 (82.3) | 1.06 (0.30 – 1.82) | **0.01** |
| Yes | 26 (17.7) | 1.08 (-1.20 – 3.35) | 0.33 |
| **Comorbidities^a^** |  |  |  |
| 0 | 51 (34.7) | 2.14 (0.90 – 3.37) | **0.001** |
| 1+ | 96 (65.3) | 0.34 (-0.50 – 1.19) | 0.42 |
| **Physical activity** |  |  |  |
| No | 114 (77.6) | 0.28 (-0.56 – 1.12) | 0.76 |
| Yes | 33 (22.5) | 1.94 (0.83 – 3.06) | **0.001** |

## Supplementary Table S7: Subgroup analysis for the association between World Cancer Research Fund/American Institute of Cancer Research (WCRF/AICR) score and overall Depression and Anxiety scores (HADS) in multivariable models.

Abbreviations: WCRF/AICR = World Cancer Research Fund/American Institute of Cancer Research, HADS = The Hospital Anxiety and Depression Scale, BMI = Body mass index, kg/m² = kilograms per square metre.

*Adjusted for age (continuous), total energy intake (continuous), education level (high school or less / TAFE or diploma or University), marital status (partner / no partner), smoking (Never / former / current), and comorbidities (0 / 1+). Stratified factors were removed from the models, estimating their subgroup analysis

^a^ Based on the Charlson Comorbidity Index score

## Supplementary Table S8: Spearman correlation test between the continuous total scores of MDS, WCRF/AICR, and EHLI at baseline (N=147).

| **Spearman Correlation Coefficients**  **p-values under H₀: ρ = 0** | | | |
| --- | --- | --- | --- |
|  | **MDS** | **WCRF/AICR** | **EHLI** |
| **MDS** | 1.0000 | 0.20  0.01 | 0.30  0.0002 |
| **WCRF/AICR** | 0.20  0.01 | 1.0000 | 0.77  < 0.0001 |
| **EHLI** | 0.27  0.0009 | 0.77  < 0.0001 | 1.0000 |

Abbreviations: MDS = Mediterranean Diet Score, WCRF/AICR = World Cancer Research Fund/American Institute of Cancer Research, EHLI = Extended Healthy Lifestyle Index.

Note: Values represent Spearman correlation coefficients (ρ), with corresponding p-values shown below each coefficient. P-values indicate the probability under the null hypothesis (H₀: ρ=0)

## Supplementary Table S9: Weighted kappa test of agreement between tertiles of MDS, WCRF/AICR, and EHLI at baseline (N=147).

| **Weighted kappa statistics (κ)**  **p-values under H₀: agreement is due to chance (Pr > \|z\|)** | | | |
| --- | --- | --- | --- |
|  | **MDS tertiles** | **WCRF/AICR tertiles** | **EHLI tertiles** |
| **MDS tertiles** |  | 0.13  0.0466 | 0.27  < 0.0001 |
| **WCRF/AICR tertiles** | 0.13  0.0466 |  | 0.54  < 0.0001 |
| **EHLI tertiles** | 0.27  < 0.0001 | 0.54  < 0.0001 |  |

Abbreviations: MDS = Mediterranean Diet Score, WCRF/AICR = World Cancer Research Fund/American Institute of Cancer Research, EHLI = Extended Healthy Lifestyle Index.

Note: Values represent weighted kappa statistics (κ), with corresponding p-values (Pr > |z|) shown below each statistic. P-values indicate the probability under the null hypothesis (H₀) that the observed agreement is due to chance
